# Comparative Genomics of an Emerging Amphibian Virus

**DOI:** 10.1534/g3.115.023762

**Published:** 2015-10-30

**Authors:** Brendan Epstein, Andrew Storfer

**Affiliations:** School of Biological Sciences, Washington State University, Pullman, Washington 99164

**Keywords:** Ranavirus, *Ambystoma tigrinum* virus, range expansion

## Abstract

Ranaviruses, a genus of the Iridoviridae, are large double-stranded DNA viruses that infect cold-blooded vertebrates worldwide. Ranaviruses have caused severe epizootics in commercial frog and fish populations, and are currently classified as notifiable pathogens in international trade. Previous work shows that a ranavirus that infects tiger salamanders throughout Western North America (*Ambystoma tigrinum* virus, or ATV) is in high prevalence among salamanders in the fishing bait trade. Bait ATV strains have elevated virulence and are transported long distances by humans, providing widespread opportunities for pathogen pollution. We sequenced the genomes of 15 strains of ATV collected from tiger salamanders across western North America and performed phylogenetic and population genomic analyses and tests for recombination. We find that ATV forms a monophyletic clade within the rest of the Ranaviruses and that it likely emerged within the last several thousand years, before human activities influenced its spread. We also identify several genes under strong positive selection, some of which appear to be involved in viral virulence and/or host immune evasion. In addition, we provide support for the pathogen pollution hypothesis with evidence of recombination among ATV strains, and potential bait-endemic strain recombination.

Emerging infectious diseases are increasingly appreciated as a leading health concern for humans, wildlife, and economically important agricultural populations (Daszak *et al.* 2000; Smith *et al.* 2009). Indeed, pathogens are now listed as a leading cause of species’ declines and extinctions (De Castro and Bolker 2005; Smith *et al.* 2006). Ranaviruses, a genus of the Iridoviridae, are globally-distributed pathogens of amphibians, reptiles and commercial fish species (Chinchar 2002; Miller *et al.* 2011). These large, double-stranded DNA viruses are considered emerging due to increases in incidence and geographic range over the last 30 years (Daszak *et al.* 2000; Chinchar 2002; Miller *et al.* 2011).

Ranaviruses are now classified as notifiable pathogens in international trade because of their effects on commercial and wildlife populations (Schloegel *et al.* 2010). Pathogen pollution is of particular concern, whereby non-native ranavirus strains are introduced into host populations with which they have no evolutionary history, potentially leading to large scale epizootics (Cunningham *et al.* 2003). Phylogenetic analyses provide several lines of evidence for host switching events among the 10 completely sequenced *Ranavirus* genomes (Jancovich *et al.* 2010; Abrams *et al.* 2013). These analyses suggest that TFV (tiger frog virus) and GIV (grouper iridovirus) are likely strains of the geographically-distant FV3 (frog virus 3) and SGIV (Singapore grouper iridovirus), respectively (Chinchar *et al.* 2011). In addition, strains isolated from different vertebrate classes, such as STIV (soft-shelled turtle iridovirus; Reptilia) and FV3 (Amphibia) are very similar in genome organization, and, like RGV (Lei *et al.* 2012), both have truncated versions of the viral homolog of eukaryotic translational initiation factor 2α [*vIF-2*α (Huang *et al.* 2009; Tan *et al.* 2004)]. Additional phylogenetic analyses, combined with comparisons of genomic organization, suggest that the most recent common ancestor of all ranaviruses was a strain that infected fish (Jancovich *et al.* 2010; Chinchar *et al.* 2011). Evidence of a fish–amphibian host switch comes from the strong collinearity of EHNV (epizootic hematopoietic necrosis virus; isolated from rainbow trout) and ATV (*Ambystoma tigrinum* virus, isolated from salamanders). A possible mechanism for host switching among vertebrate classes was inferred from evidence of positive selection on 12 ranavirus genes; six of these genes were apparently acquired as a result of host switches (Abrams *et al.* 2013).

The broad host range and propensity for host switching among ranavirus strains has led to widespread concerns surrounding the potential effects of pathogen pollution. Two ranavirus strains that infect amphibians have received considerable attention in this regard. FV3 and FV3-like strains, which have a broad host range, are commonly found when international shipments of bullfrogs are tested using PCR-based methods (Schloegel *et al.* 2009). In addition, ATV—a ranavirus that infects tiger salamanders across the North American cordillera (Jancovich *et al.* 1997, 2003, 2005)—has been found to have > 80% prevalence in tiger salamanders in bait shops across Arizona, Colorado, and New Mexico (Picco and Collins 2008). ATV causes seasonally recurrent epidemics, primarily in larval tiger salamander populations that inhabit natural and human-made ponds throughout western North America (Jancovich *et al.* 1997, 2005; Brunner *et al.* 2004; Figure 1). Phylogeographic analyses suggest that long-distance ATV dispersal follows highways that are known to be used for transporting bait (Jancovich *et al.* 2005), and angler surveys confirmed release of unused live bait salamanders into lakes and ponds (Picco and Collins 2008). Thus, extensive opportunities exist for pathogen pollution by ATV.

**Figure 1:**
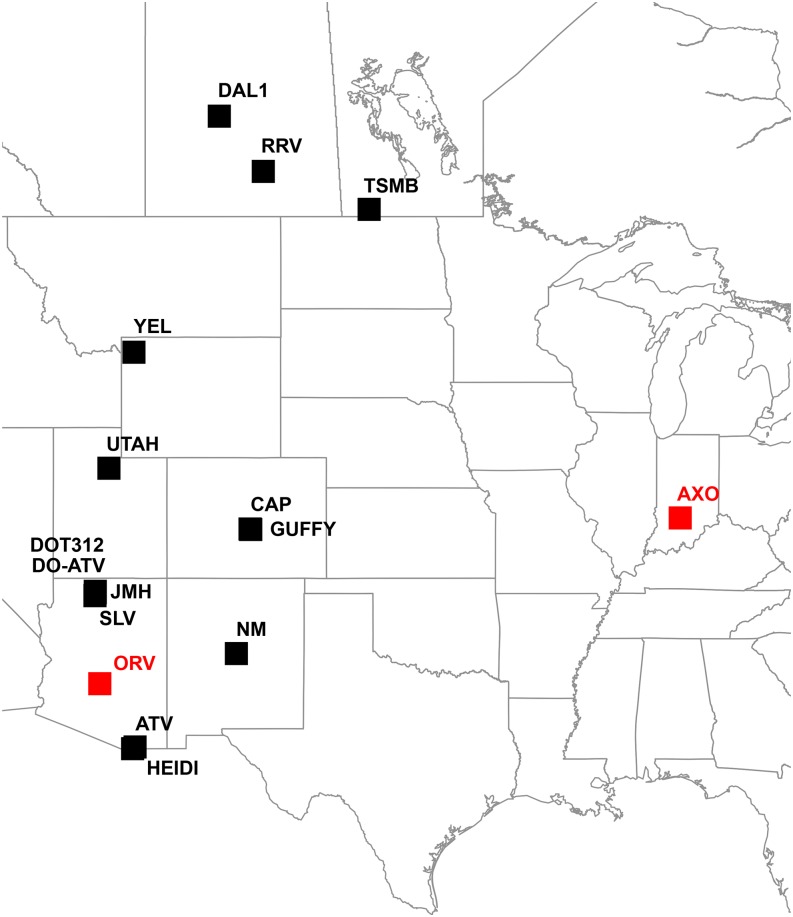
Locations of the tiger salamander populations from which the strains of *Ambystoma tigrinum* virus (ATV) sequenced in this study were obtained. Strains marked in red were collected in a bait shop (ORV) and from a captive axolotl colony (AXO), and hence their location of origin was not known.

A reference genome of ATV has been sequenced (Jancovich *et al.* 2003), and phylogenetic analysis of ATV strains (using DNA sequences comprising about 2% of the genome) throughout the western United States suggests monophyly (Jancovich *et al.* 2005; Storfer *et al.* 2007). However, ATV appears to evolve differently in different host populations. That is, of nine genes (ORFs) that have a putative function in host immune evasion and/or viral virulence (Jancovich *et al.* 2003), two were under positive selection among tiger salamander host populations (Ridenhour and Storfer 2008). Indeed, there is also evidence of coevolution of ATV and tiger salamander hosts as comparative phylogenetic analyses show strong concordance between host and virus phylogenetic trees (Storfer *et al.* 2007). Thus, it is important to understand genomic variation among ATV strains to help assess the potential effects of pathogen pollution, as coevolutionary dynamics of hosts and pathogens could be disrupted by introduction of non-native bait strains.

Here, we sequenced the complete genomes of 15 ATV native and bait-associated strains across a broad geographic area to assess genomic diversity, identify genes that are experiencing strong positive selection, and to test for evidence of recombination. Recombination has been reported to occur among ranaviruses (*e.g.*, Abrams *et al.* 2013), but has not been verified or quantified. A previous study showed that an ATV strain isolated from a bait shop had significantly greater virulence than endemic, coevolved strains (Storfer *et al.* 2007). Thus, recombination among non-native and native ATV strains could enhance virulence, leading to severe epizootics and possibly local extirpation. We also conducted phylogenetic analyses of ATV in comparison to other *Ranavirus* genomic sequences to provide a rigorous test of monophyly *vs.* a pattern of nonmonophyly resulting from repeated host-switches, as well as timing of the emergence of ATV to assess human involvement in its geographic spread.

## Materials and Methods

### Strain collection

The genome sequences of 11 previously sequenced *Ranavirus* strains were obtained from GenBank: *Ambystoma tigrinum* virus (ATV), *Andrias davidianus* ranavirus (ADRV), common midwife toad virus (CMTV), tiger frog virus (TFV), frog virus 3 (FV3), *Rana grylio* virus (RGV), soft-shelled turtle iridovirus (STIV), European sheatfish virus (ESV), epizootic hematopoietic virus (EHNV), grouper iridovirus (GIV), and Singapore grouper iridovirus (SGIV) (Table 1). The genome sequences of 15 additional *Ranavirus* isolates collected from North America between 1997 and 2003 are described for the first time here (Table 1). Reads and assemblies for these 15 strains have been deposited at NCBI in BioProject PRJNA257291.

**Table 1:** Locations, collection dates, hosts, and number of reads obtained for strains included in this study

Strain	Full Name	Location	Year	Host	Reference	NCBI Accession	Assembly Size	Reads
Previously sequenced								
ATV	*Ambystoma tigrinum* virus	Santa Cruz, AZ (31°24’ N, 110°27’W)	1995	*Ambystoma tigrinum*	Jancovich *et al.* 2003	NC_005832.1	106,332	
ADRV	*Andrias davidianus* ranavirus	Shanxi Province, China	2010	*Andrias davidianus*	Jiang *et al.* 2011; Wang *et al.* 2014; Geng *et al.* 2011	KF033124.1	106,719	
TFV	Tiger frog virus	Nanhai, Guangdong, China	∼1999	*Rana tigrina rugulosa*	He *et al.* 2002	AF389451.1	105,057	
EHNV	Epizootic hematopoietic necrosis virus	Lake Nillahcootie, Benalla, Australia	1984	*Percia fluviatilis*	Langdon *et al.* 1986; Jancovich *et al.* 2010	FJ433873.1	127,011	
FV3	Frog virus 3	Wisconsin / Minnesota	1962	*Rana pipiens*	Tan *et al.* 2004; Granoff and Came 1965	AY548484.1	105,903	
CMTV	Common midwife toad virus	Picos de Europa Nat’l Park, Spain	2007	*Alytes obstetricans*	Balseiro *et al.* 2009; Mavian *et al.* 2012a	JQ231222.1	106,878	
STIV	Soft-shelled turtle iridovirus	Shenzhen, China	1997	*Trionyx sinensis*	Chen *et al.* 1999; Huang *et al.* 2009	EU627010.1	105,890	
RGV	*Rana grylio* virus	Wuhan, Hubei, China	1995	*Rana grylio*	Zhang *et al.* 2001; Lei *et al.* 2012	JQ654586.1	105,791	
ESV	European sheatfish virus	Germany	1989	*Silurus glanis*	Mavian *et al.* 2012b	JQ724856.1	127,732	
GIV	Grouper iridovirus	Taiwan	2000 or earlier	*Epinephelus awoara*	Lai *et al.* 2000; Tsai *et al.* 2005	AY666015.1	139,793	
SGIV	Singapore grouper iridovirus	Singapore	1998	*Epinephelus tauvina*	Qin *et al.* 2001; Song *et al.* 2004	AY521625.1	140,131	
Sequenced here								
AXO		Axlotl colony, Indiana University	2001	*Ambystoma mexicanum*	Jancovich *et al.* 2005	KR075872	105,504	565,445
CAP		Cap Mountain Pond, CO (38°38’N, 105°24’W)	2000	*Ambystoma tigrinum*	Jancovich *et al.* 2005	KR075886	106,004	543,964
DO-ATV		“Doughnut Tank”, AZ (36°25’20’’N, 105°12’40’’W)	2000	*Ambystoma tigrinum*	Ridenhour and Storfer 2008	KR075885	105,936	582,711
DOT312		“Doughnut Tank”, AZ (36°25’20’’N, 105°12’40’’W)	2003	*Ambystoma tigrinum*	J. Brunner, unpublished data	KR075883	107,829	726,469
GUFFY		Guffy Pond, CO (36°37’N, 105°22’W)	2001	*Ambystoma tigrinum*	Jancovich *et al.* 2005	KR075882	106,437	931,263
HEIDI		“Heidi Tank”, AZ (31°20’35’’N, 110°33’51’’W)	2000	*Ambystoma tigrinum*	Ridenhour and Storfer 2008	KR075873	106,230	735,634
JMH		“Joe’s Mud Hole”, AZ (36°34’N, 112°12’W)	2000	*Ambystoma tigrinum*	Jancovich *et al.* 2005	KR075881	106,380	659,985
NM		Paige Well, NM, 33° 06’ N 107° 39’ W	2004	*Ambystoma tigrinum*	Ridenhour and Storfer 2008	KR075880	107,371*^a^*	554,072
ORV		Phoenix, AZ bait shop	1998	*Ambystoma tigrinum*	Jancovich *et al.* 2005	KR075874	106,018	1,311,233
SLV		Snipe Lake, AZ (36°31’N, 112°12’W)	2000	*Ambystoma tigrinum*	Jancovich *et al.* 2005	KR075878	106,722	487,984
TSMB		Boissevain, MB, Canada (49°14’N, 100°10’W)	1998	*Ambystoma tigrinum*	Jancovich *et al.* 2005	KR075875	106,526	448,595
UTAH		Lake Desolation, UT (40°39’N, 111°36’W)	1998	*Ambystoma tigrinum*	Jancovich *et al.* 2005	KR075877	106,198	625,978
YEL		Yellowstone National Park, USA	2003	*Ambystoma tigrinum*	J. Eastman and A. Storfer, unpublished data	KR075876	105,922	654,185
DAL1		Dalmeny SK, Canada (52°20’N, 106°45’W)	2000	*Ambystoma tigrinum*	Jancovich *et al.* 2005	KR075884	105,354	667,658
RRV		Regina, SK, Canada (50°30’N, 104°49’W)	1997	*Ambystoma tigrinum*	Jancovich *et al.* 2005	KR075879	106,971	539,385

a Draft assembly with two contigs; all other assemblies contain only one contig.

### DNA extraction

ATV strains were isolated from infected tiger salamanders at each of the 15 localities by first freezing the entire salamander at –70°. Then, each salamander was homogenized in Eagle’s minimum essential medium (MEM), and the slurry was filtered in preparation for viral cell culture. Cell culture protocols for replication of ATV have been standardized (Jancovich *et al.* 1997, 2001); the 15 virus isolates to be used herein all produced cytopathology in cultured *Epithelioma papilloma cyprini* fish cells (Fijan *et al.* 1983) within 48–72 hr. Virus strains were triple plaque-purified and cultivated with no more than three total passages and equal number of passages per strain, because serial passages through clonal cell lines could result in viral evolution, such as adaptation to the cell line and consequent attenuation in the host (see Ebert 1999). Each strain was replicated to approximately 100 ml in volume in MEM, and viral DNA extraction followed a modified sucrose-gradient protocol (Thurber *et al.* 2009). In brief, DNA was extracted by lysing the host cells with three freeze–thaw cycles, then incubating with DNase, layering the DNA onto a 25% sucrose pad, and spinning at 16,000 rpm for 8 hr in an ultracentrifuge. The DNA was then purified by treatment with proteinase K and RNase followed by a phenol extraction.

### Sequencing, assembly, and annotation

Libraries were prepared using standard protocols and ATV strains were sequenced at the University of Idaho Genomics Resources Core using 300-bp paired-end reads on an Illumina MiSeq, resulting in a mean of 670,000 reads per strain (Table 1). The reads were cleaned with seqyclean (available from https://github.com/ibest/seqyclean) using ESTs from *Pimephales promelas* as contaminants, then overlapping read pairs were merged using Flash (v1.2.8; Magoč and Salzberg 2011). A reference-guided assembly was performed using ARC (Hunter *et al.* 2015), with both ATV and FV3 as the reference genome in separate analyses, followed by manual finishing. For most strains, the best assembly (most continuous) was obtained using SPAdes (v3.0.0; Bankevich *et al.* 2012) as the assembler, except for ORV and JMH (see Table 1), for which we used Newbler (Marguiles *et al.* 2005). We obtained assistance with the ORV and JMH assemblies from the University of Idaho Genomics Core. To correct errors in the assembly and remove any reference bias, we aligned the reads for each strain back to its assembly. Any position not covered by ≥ 10 uniquely aligning reads, and with a single base call supported by at least 80% of the uniquely aligning reads with a quality score ≥ 30, and with support on reads aligning to both strands was changed to “N.” If the aligned reads supported a different base than the assembly, the assembly was changed. We relaxed the requirement for alignment to both strands for the first and last 100 bp of the assembly, and we trimmed any remaining terminal ambiguities. The final fold coverage of the assemblies ranged from approximately 350 to 2300.

Although relatively long, paired-end reads and very high coverage should result in high accuracy, we verified the assembled sequence at seven loci, including two repetitive regions, using 50,833 bp of Sanger sequence data from 14 strains (no Sanger data were available for DOT312) (Genbank accessions EU512250-EU512270 and EU512376-EU512396; Ridenhour and Storfer 2008). These data included two variable repeat regions.

We annotated the genomes using the Rapid Annotation Transfer Tool (RATT; Otto *et al.* 2011), with ATV, FV3, and CMTV as the reference genomes, ATG as the start codon, TGA, TAA, and TAG as stop codons, no splice site correction, and no pseudogene correction. We used a custom python script to remove potentially redundant annotations (we picked the longest ORF for annotations with the same stop codon). We also removed 20–30 ORFs that were split into multiple pieces, because none of the amphibian-like ranavirus (ALRV) sequences from GenBank have introns or split features. BLASTP (v2.2.28; Camacho *et al.* 2009) was used to search Swissprot (downloaded April 9, 2014; Boeckmann *et al.* 2003) to find more descriptive annotations for the genes.

Sets of orthologous genes were identified using blastclust (Camacho *et al.* 2009) using a threshold of 80% amino acid identity across 80% of the length of both sequences; to construct the genealogy with GIV strains, we required only 60% identity across 60% of the length of both sequences. The protein sequences of orthologous genes were aligned using muscle (Edgar 2004), and the protein sequence alignment was then used to construct the nucleotide alignment. We also aligned the nucleotide sequences separately from the protein sequences and considered indels that were not a multiple of 3 bp long to be potential frameshifts. To avoid frameshift mutations being erroneously treated as nonsynonymous mutations, alignments in genes with potential frameshifts were trimmed to the last codon before the frameshift before running phylogenetic or population genomic analyses.

### Dotplots

Pairwise genome comparisons were conducted using the dnadiff program from MUMmer (Kurtz *et al.* 2004), and dotplots were drawn using custom R software (R Core Team 2014) code. Dotplots are used for visualizing structural differences between pairs of genomes: the axes are position in each genome, and a dot is placed at every location where the two genomes have similar sequence. Identical genomes appear as a diagonal line, while inversions appear as changes in the orientation of matches.

### Genealogical analyses

Genealogical analyses were conducted on the concatenated nucleotide sequences of the 52 genes that were found in all ALRV strains using BEAST (v1.8.0; Drummond *et al.* 2012) and MrBayes (v3.2.2; Ronquist *et al.* 2012). Based on model-selection tests conducted with jModelTest (v2.1.4; Darriba *et al.* 2012) to determine the most likely model of molecular evolution, we used the general time-reversible model with a gamma shape parameter (GTR + G) and four site categories. In BEAST we used a lognormal relaxed clock, empirical base frequencies, and a Bayesian skyline coalescent tree prior, on data partitioned by codon position. The tip date option was used to provide dating information; the tip dates were entered as shown in Table 1 with a precision (accuracy of collection date) of 0.5, except for EHNV, ESV, and FV3, for which we entered a precision of 2.0, and TFV for which we entered a precision of 5.0. BEAST coestimates the divergence times, substitution rates, and genealogy given the sequence data and tip dates and produces estimates of divergence times integrated over the posterior distribution of topologies (Drummond *et al.* 2006). The tip dates calibrate the divergence and rate estimates, so that the result is in absolute, rather than relative, time. Because we used a relaxed clock, we do not assume that evolutionary rates are constant across all branches; rather the rates are drawn from a lognormal distribution. Nearly all of the priors were left as default, except for the relative rate parameters for the codon positions, which were set to a finite uniform prior. Five independent runs with 200,000,000 iterations each were conducted and combined, after confirming that the results of each run were very similar, with logcombiner (Drummond *et al.* 2012). The first 10% of each run was discarded as burn-in. We confirmed that the chains converged and had sufficient sampling using tracer (Drummond *et al.* 2012). Finally, we constructed a maximum clade credible tree (MCCT) using treeannotator (Drummond *et al.* 2012) with median branch heights. A strict molecular clock was rejected by examining the distribution of the ucld.stdev parameter—the distribution did not overlap with zero. We used BEAST to construct the Bayesian skyline plot for just the ATV strains using the same methods, except that the 71 genes found in all ATV strains were included.

We also conducted two runs of MrBayes: one with the same 52 genes used for the BEAST coalescent tree analysis and another with 17 genes found in all ALRV strains plus GIV and SGIV. We used the GTR + G model of evolution with four rate categories and partitioning by codon position. MrBayes was for 12,000,000 iterations, and we verified that the standard deviation of split frequencies was < 0.01.

### PAML analyses

**Figure 2:**
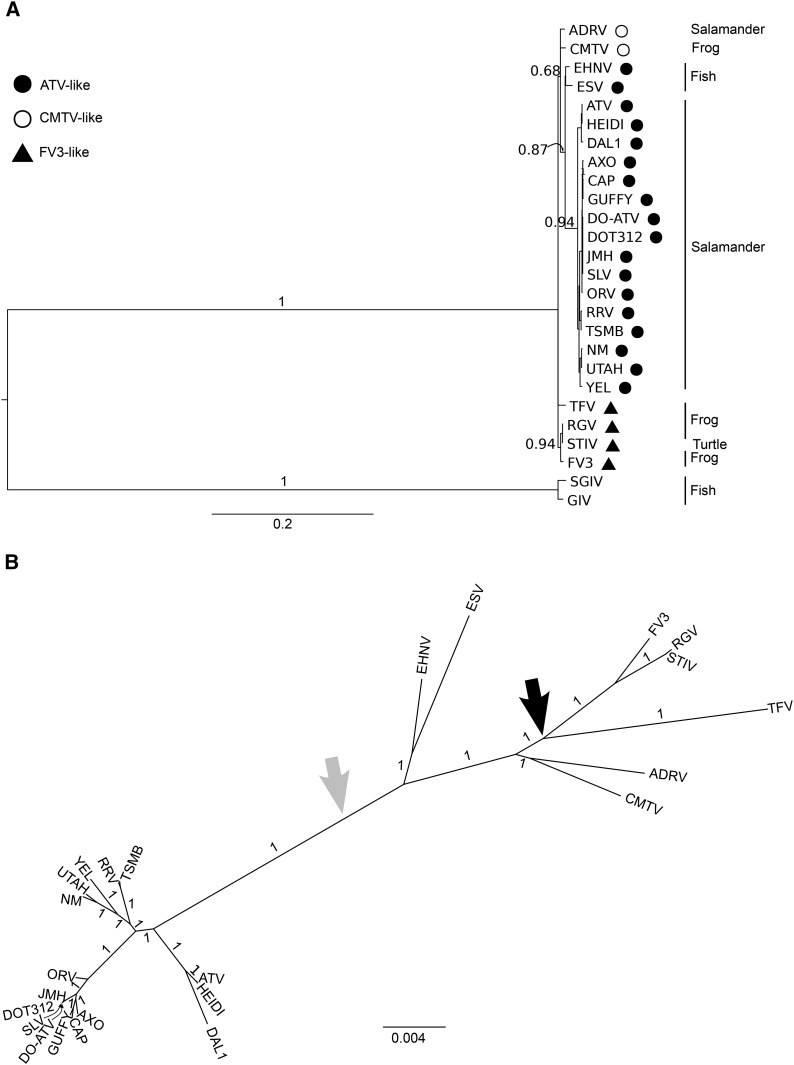
Bayesian genealogy of *Ranavirus*. (A) The genealogy constructed with MrBayes using the 17 genes found in all *Ranavirus*; for the sake of clarity, Bayesian posterior support values (edge labels) are shown only for the older nodes. (B) Relationships constructed using 52 genes found in all amphibian-like ranavirus (ALRV) strains using MrBayes. (A) was rooted using Singapore grouper iridovirus (SGIV) and grouper iridovirus (GIV) as a outgroup. The large, black arrow in (B) shows the location of this outgroup; due to a polytomy in (A), the exact location of the root branch in (B) is ambiguous, although the relative ancestry of the ATV and European sheatfish virus/epizootic hematopoietic virus (ESV/EHNV) clades are not affected by the ambiguity. A genealogy of the ALRV strains constructed using BEAST had an identical unrooted topology and qualitatively similar branch lengths and support values, but with a different root placement in the maximum clade credible tree (shown with a light gray arrow). The closed circle, open circle, and triangle indicate an ATV-like, common midwife toad virus (CMTV)-like, or frog virus 3 (FV3)-like genome structure, respectively. Host taxa are indicated to the right of the tree on (A).

We searched for evidence of positive selection using PAML (v4.7; Yang 2007), which identifies genes with elevated ratios of nonsynonymous to synonymous substitutions (ω). We ran three analyses on the ATV clade (other strains were excluded): site tests comparing a model that included neutral sites and sites under purifying selection (model 8A; specified by setting NSsites = 8, fix_omega = 1, omega = 1) to a model that also allowed positive selection (model 8; NSsites = 8, fix_omega = 0); and two sets of branch-site tests comparing a model with neutral sites and purifying selection at all branches (model 2A; NSsites = 2, model = 2, fix_omega = 1, and omega = 1) to a model that also allows positive selection on particular branches and sites (model 2; NSsites = 2, model = 2, and fix_omega = 0). The branch leading to DAL1 was tested because it appears to be geographically distant from its closest relatives (Figure 1 and Figure 2), and the branches leading to UTAH, ORV, and AXO were tested because these are human-associated or bait strains (Ridenhour and Storfer 2008; J. Eastman and A. Storfer, unpublished data). To identify genes with strong positive selection, we conducted a likelihood ratio test with 1 degree of freedom and divided the p-value by two, as recommended in the PAML manual. In all cases, we set PAML to remove sites with indels or ambiguities, used the branch lengths in the MrBayes genealogy as the starting point, did not assume a molecular clock, and estimated codon frequencies from base frequencies. Only the 93 ORF clusters with ≥ 11 ATV strains and ≥ 15 codons were included. To avoid problems with lack of convergence, we ran both the site and branch-site models three times with different initial values, and chose the runs with the least negative log-likelihood. ORF cluster 94 was removed from the site test results because PAML indicated that every codon was under positive selection in spite of having only two variable sites.

### Population genetic analysis

To characterize the nucleotide diversity of ATV, we calculated Watterson’s θ (θ_W_; Watterson 1975), π (θ_π_; Kimura 1968) Tajima’s D (D_T_; Tajima 1989), and the McDonald-Kreitman test statistics (MK test; McDonald and Kreitman 1991) using custom C++ programs based on libsequence (Thornton 2003). We include only biallelic sites without ambiguities. Watterson’s θ and π are coalescent-based estimators of the population mutation rate (θ = 2N_e_μ) assuming standard neutral conditions and infinite sites or infinite alleles, respectively, that are useful as a sample-size adjusted measurement of nucleotide diversity. Under the infinite alleles and infinite sites models, it is expected that all polymorphisms are biallelic: overall, 95% of polymorphic sites have two alleles, and if five ORF clusters (clusters 12, 66, 82, 86, and 95) that were challenging to align are excluded, > 99% of polymorphic sites have two alleles. Thus, there is no evidence for a serious violation of the infinite sites or alleles models. Tajima’s D summarizes the allele frequency spectrum, and can serve as an indicator of demographic events that result in changes in population size or selective sweeps. Tajima’s D is negative after recovery from a selective sweep or during rapid population expansion (negative D values can also result from background purifying selection), positive under balancing selection or population decline, and 0 under the standard neutral model. We used only genes with data from ≥ 11 of the ATV strains; Tajima’s D was reported only if there were ≥ 3 SNPs in a gene. The MK test compares the ratio of nonsynonymous polymorphism: synonymous polymorphism to the ratio of nonsynonymous divergence: synonymous divergence; excess levels of divergence are indicative of positive selection. For the MK test, we used the ESV/EHNV clade as an outgroup. A modified version of the MK test has been suggested for viral datasets with large numbers of multi-allelic sites (Bhatt *et al.* 2010); however, as discussed above, our dataset contained few multi-allelic sites.

### Recombination analysis

We tested for the presence of recombination in the ATV clade using the software packages RDP4 (v4.36; Martin *et al.* 2010) and Rbrothers (v0.0.1; Irvahn *et al.* 2013). The tests in RDP4 search an alignment for positions where there is a change in relative sequence similarity among strains; RDP4 then attempts to combine the results from multiple methods into a single set of results. Rbrothers searches for changes in phylogeny along an alignment, but does not attempt to identify specific events; rather, it estimates the most likely number of recombination events. We first aligned the ATV clade genomes using mugsy (Angiuoli and Salzberg 2011), and then stitched the aligned blocks together using ATV as a reference, dropping alignment blocks that did not contain ATV (these regions were small, contained few strains, and tended to be poorly aligned). For RDP4, we use the RDP (Martin and Rybicki 2000), Geneconv (Padidam *et al.* 1999), BootScan (Martin *et al.* 2005), MaxChi (Smith 1992), Chimaera (Posada and Crandall 2001), SiSscan (Gibbs *et al.* 2000), and 3Seq (Boni *et al.* 2007) methods with the default settings during both the primary scan (identification of recombination) and secondary scan (verification) phases. Although these methods differ in algorithmic and statistical details, all search an alignment for positions where a change in relative sequence similarity occurs, using three or four sequences at a time. The defaults include a p-value threshold of 0.05 and a Bonferroni correction for multiple comparisons; due to the size of the dataset, we did not select the option to require phylogenetic evidence for recombination events. The RDP4 package attempts to group similar signals of recombination into single events that may represent ancestral recombination events, and also attempts to identify the source (“minor parent”) of the recombined region. For Rbrothers, which uses a dual multiple change-point model (Minin *et al.* 2005) to identify positions where the phylogeny constructed using all sequences changes, we used a 4000-bp sliding window (2000-bp step) to generate the candidate trees, and ran the chain for 41,000,000 iterations, including 1,000,000 iterations of burn-in. As recommended by Irvahn *et al.* (2013), the analysis was repeated for several “lambda_prior” (expected number of breakpoints) values: 1, 5, 10, and 20. Rbrothers does not attempt to characterize the details of recombination events, but rather tries to determine the most likely number of events.

To estimate the population scaled recombination rate ρ = 2N_e_r (where N_e_ = effective population size and r = recombination rate), we used the interval program in the LDHat package (v2.2; Auton and McVean 2007). The interval program identifies recombination by fitting a coalescent model with recombination and mutation to the sequence data using a Markov Chain Monte Carlo approach. We used the same whole genome alignment as described above and ran interval for 10,000,000 iterations, sampling every 2000 after discarding the first 100,000 iterations as burn-in. Both crossing-over mode and gene-conversion mode were used, with gene conversion tract lengths 25, 50, 100, 500, and 1000; and with block penalties 0, 5, 10, 20, 35, and 50. Before running interval, we removed sites that were present in less than 80% of the strains (approximately 1% of the alignment) or with a minor allele frequency < 0.2.

### Data availability

All reads, assemblies, and annotations generated in this study have been deposited in GenBank under BioProject PRJNA257291.

## Results

### Genome size and structure

**Figure 3:**
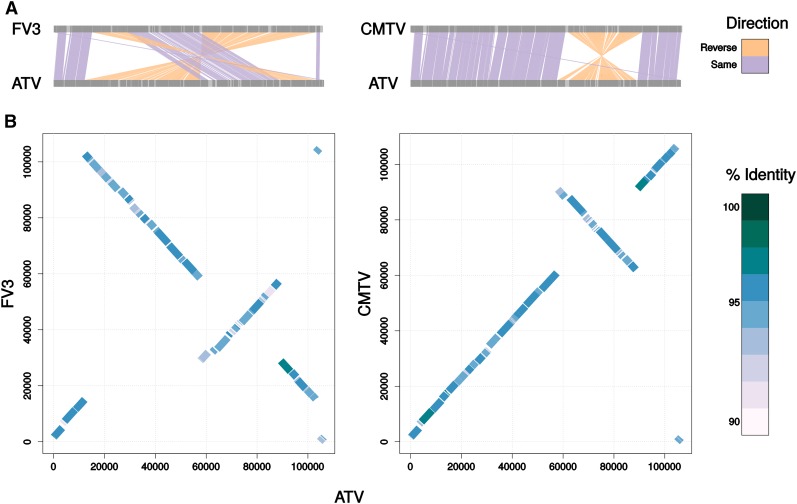
Comparison of the three genome structures observed in the amphibian-like *Ranavirus* strains. (A) Synteny plots comparing ATV and FV3, and ATV and CMTV. (B) The same alignments represented as dotplots, with ATV on the x-axis. Compared to ATV, CMTV has an inversion (60–90 kb in ATV), and FV3 has an additional inversion encompassing most of the genome.

The lengths of the reference-guided genome assemblies ranged from 105,354 to 107,371 bp (Table 1), very similar to the reference sequence, which is 106,332 bp long (Jancovich *et al.* 2003). The mean depth of coverage was at least 350 for all strains, which is far in excess of the coverage necessary to obtain accurate base calls. In addition, 50,833 bp of Sanger sequencing data from seven regions, including two repetitive regions, revealed only 20 single base errors (assuming all mismatches between Sanger sequencing and assemblies are errors in the assemblies)—an error rate of only 0.04%—and no structural errors. Because Sanger sequencing has errors too, and the sequencing data were obtained 10 years ago, the true error rate may be less than 0.04%. The number of putative ORFs per genome ranged from 95 to 110. There was little variation in gene content with ≤ 3 unique ORFs per strain, and 75% of the genes found in the ATV reference genome (Jancovich *et al.* 2003) were also found in all the 15 strains sequenced here (see Supporting Information, File S1, Table S2), resulting in 71 ORF clusters in the ATV core genome. The core genome of all the ALRVs (all the strains included in this study except GIV and SGIV) comprised 52 ORF clusters, similar to previous results from Jancovich *et al.* (2010). Also consistent with previous studies, we found that CMTV (common midwife toad virus; Mavian *et al.* 2012a) has an inversion relative to ATV; and FV3, RGV (*Rana grylio* virus), TFV, and STIV have an additional inversion (Figure 3) (Stöhr *et al.* 2015). However, the other genomes were collinear with ATV, and there was no evidence for large-scale inversions or large indels. The ATV strains were also very similar in nucleotide sequence: genome-wide pairwise nucleotide identity ranged from 98.3 to > 99.9%, with a mean of 99.1%. Pairwise identity between ATV strains and other strains ranged from 95.4 to 97.0%, with a mean of 96.2%. Despite the similarity in gene content, there were three genes present in all ATV strains, but absent from all other strains: ORF clusters 84, 85, and 88. Interestingly, ORF cluster 84, a potential serum response factor-binding protein, shows evidence of positive selection (see below); the functions of clusters 85 and 88 are not known and they did not show significant evidence for positive selection by any of our tests.

### Genealogical relationships

We constructed a Bayesian genealogy for ALRV using BEAST (Drummond *et al.* 2012) and MrBayes (Ronquist *et al.* 2012) with the concatenated sequences of the core genome. Regardless of the set of genes or software used, strains of ATV formed a well-supported monophyletic clade (Figure 2). Using the dates strains were collected, the age of the ATV clade was estimated at 545 years (20–5672 years is the 95% highest posterior density range) and the age of the root of the tree—the common ancestor of all ALRV strains sampled here—was estimated at 1526 years (53–16,524).

**Figure 4:**
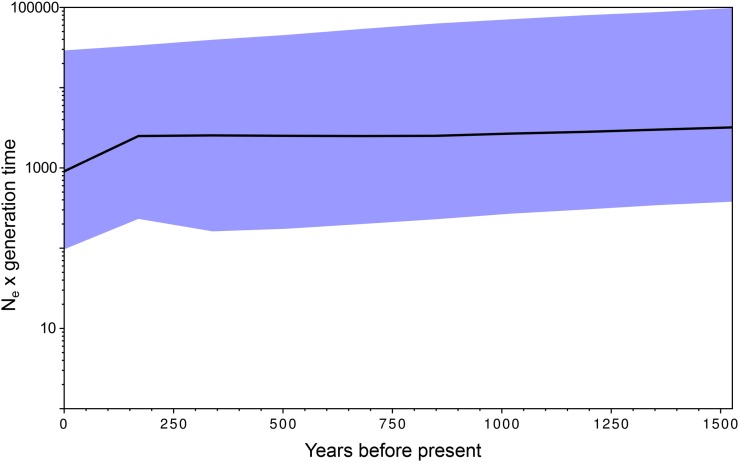
Bayesian skyline plot of the Amphibian-like Ranaviruses. The y-axis is proportional to effective population size and the x-axis is time from collection of the samples (around the year 2000 for most strains). The median estimate is given by the black line and the 95% confidence intervals are shaded in blue.

To see whether a signal of recent population expansion in ATV or ALRV could be detected, we constructed a Bayesian skyline plot (Drummond *et al.* 2005) for the ALRV group using BEAST. Effective population sizes over the last several hundred years are stable relative to the size of the confidence intervals, although the point estimate shows a decline over the last 100 years (Figure 4). A plot constructed for just the ATV group also showed a stable effective population size (Figure S1).

### Population genetic diversity

**Figure 5:**
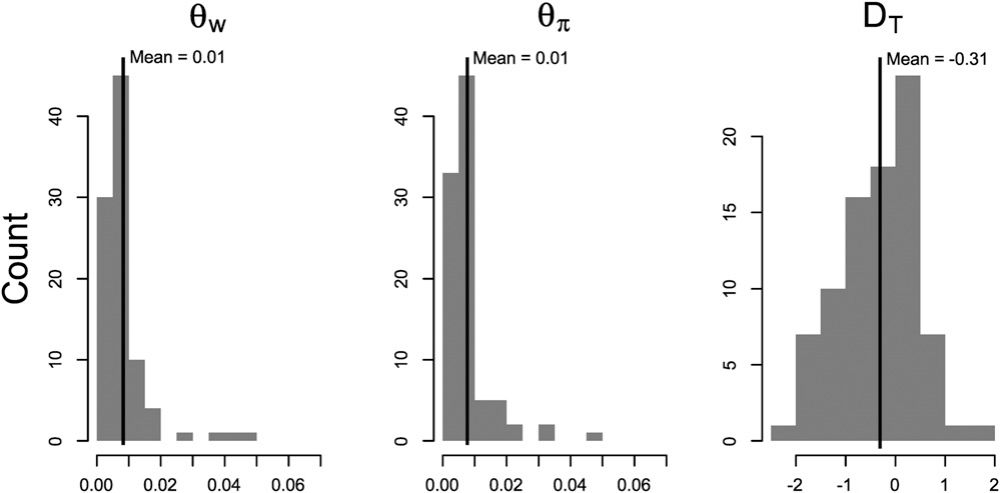
Distributions and means for population genetic statistics. Statistics were calculated for the ATV clade on genes with at least 11 strains present. θ_W_ and θ_π_ are measures of nucleotide diversity. Tajima’s D (D_T_) describes the shape of the allele frequency spectrum: the neutral expectation is 0, while selective sweeps or population expansion result in negative values and balancing selection or population contraction result in positive values.

To characterize the nucleotide diversity in ATV, we calculated population genetic statistics for the 93 ORF clusters found within at least 11 strains in the ATV clade (Figure 5). On average, across all clusters, θ_W_, the average number of segregating sites per bp, and θ_π_, the average number of pairwise differences per bp, were both 0.01. Tajima’s D (calculated for 85 genes that had ≥ 3 SNPs) was skewed negative but had a mean near 0; the 95% confidence interval obtained by bootstrapping over genes 200 times was –0.47 to –0.15.

### Selection

We searched for ORF clusters under strong positive selection within the ATV clade by testing for elevated ratios of nonsynonymous to synonymous mutations (ω) with PAML (Yang 2007) site and branch-site tests. We also tested for genes with evidence of adaptive divergence between the ATV and ESV/EHNV clades with the McDonald-Kreitman (MK) test. Eight ORF clusters (Table 2) showed significant evidence of positive selection, after a sequential Bonferroni correction for multiple comparisons (eight clusters by site tests, and two clusters by branch-site tests). Sixteen additional clusters also showed a significant signature of positive selection (*P* < 0.05) if no multiple test correction was performed (Table 2). Several of these genes are potentially involved in interactions with the host immune system or replication machinery; for example, ORF cluster 84 may be a cell cycle regulator. However, some of the clusters with the strongest evidence of positive selection had alignments with many indels, which makes alignment challenging. Two more gene clusters, 93 (3-β-hydroxy-D 5-C27-steroid oxidoreductase-like protein) and 95 (no confident annotation, but shows homology to collagen-like protein 1), showed strong evidence of positive selection in PAML site tests (*P* ≤ 0.0001). ORF cluster 95 may make up part of the viral surface such as envelope proteins; and ORF cluster 93 is involved in suppression of host immunity (Chinchar 2002; Miller *et al.* 2011). However, both of these genes included several sequences with mutated start codons or frameshift mutations that likely result in a nonfunctional product. After removing these sequences, cluster 93 no longer showed evidence for positive selection. On the other hand, cluster 95, while now having fewer than our 11-strain cutoff for running PAML tests, still showed evidence for selection by site tests (*P* < 0.0001) and branch-site tests (*P* = 0.0001). Overall, the PAML analysis indicated that most nonsynonymous sites in most genes are neutral or under purifying selection in the ATV clade: ω << 1 for most genes (Figure S2).

**Table 2:** Genes with evidence for positive selection in *Ambystoma tigrinum* virus (ATV) (omega for positively selected codons, p-value).

Gene Cluster	ATV Gene	Site Test	Branch-Site DAL1	Branch-Site Human-Associated	MK Test	Annotation
1	ATV_AAP33221.1	0.04, 2.0				DNA-dependent RNA polymerase, subunit a
2	ATV_AAP33249.1	0.05, 2.4				Unknown
6	ATV_AAP33184.1	0.007, 11.2				NTPase
8	ATV_AAP33245.1				0.02	Ribonucleoside diphosphate reductase
9	ATV_AAP33230.1				0.01	Myristylated membrane protein
15	ATV_AAP33229.1	0.002, 11.6				Helicase
16	ATV_AAP33190.1				0.01	Immediate early protein (ICP-46)
23	ATV_AAP33268.1				0.03	Unknown
33	ATV_AAP33200.1	0.005, 42.4				Immediate early protein (ICP-18)
38	ATV_AAP33188.1				0.05	Unknown
40	ATV_AAP33254.1	0.009, 6.8				Unknown
54	ATV_AAP33207.1	0.005, 5.2				Unknown
62	ATV_AAP33247.1	< 0.0001*^a^*, 11.0			0.04	Unknown
63	—	0.02, 68.9				Unknown
65	ATV_AAP33212.1	0.0003*^a^*, 22.2				Unknown protein with homology to transmembrane proteins
66	ATV_AAP33240.1	< 0.0001*^a^*, 16.8		0.004; 81.1		Neurofilament triplet ^1^H-like protein
67	ATV_AAP33256.1	< 0.0001*^a^*, 5.6	0.02, 30.7			Unknown protein with homology to SAP domain-containing protein
73	—	0.02, 7.1				Unknown
75	ATV_AAP33189.1	0.0005*^a^*, 17.8		0.002, 1.0		Unknown
82	ATV_AAP33252.1			0.002, 93	0.001	Neurofilament triplet H1-like protein
84	ATV_AAP33250.1	< 0.0001*^a^*, 9.3	< 0.0001*^a^*, NA	< 0.0001*^a^*, 218		Unknown protein with homology to serum response factor-binding protein 1
89	ATV_AAP33261.1	0.03, 13.6				Unknown protein with homology to serine/threonine kinase protein
90	—	0.0001*^a^*, 16.1				Unknown
95*^b^*	ATV_AAP33253.1	<0.0001*^a^*, 4.3	0.0001*^a^*, NA			Unknown protein with homology to collagen-like protein 1

Only significant (*P* < 0.05) results are shown. In some cases PAML listed the omega value as 999, this indicates that the estimate of K_s_ was zero; we marked the omega for these genes as NA. The ESV/EHNV clade was used as the outgroup for the MK test.

a Significant (*P* < 0.05) after sequential Bonferroni correction.

b Cluster 95 contains fewer than 11 strains after removal of nonfunctional sequences.

### Recombination

An exploratory analysis of recombination, consisting of seven tests (RDP, Geneconv, Recscan/Bootscan, SiSscan, Maxchi, Chimaera, and 3Seq) implemented in the RDP4 package (Martin *et al.* 2010), a method implemented in Rbrothers (Irvahn *et al.* 2013), and a method implemented in the interval program of the LDHat package (Auton and McVean 2007), all support evidence of recombination within the ATV clade. RDP4 detected 74 events total (best-supported 20% of events are in Table S1), while the number of recombination events detected by Rbrothers varied by parameter settings (Table 3). Using LDHat [57], our point estimates for the population scaled recombination rate (ρ = 2N_e_r) ranged from 0.00031 to 0.00033 per nucleotide, depending on parameter settings. The ratio of θ/ρ, which indicates the importance of mutation relative to recombination, was approximately 30–32, indicating that recombination, while present, typically causes substitutions much less frequently than mutation. It should be noted, however, that the mechanism of recombination among *Ranavirus* strains is not known, and so LDHat may not be able to accurately model the recombination process or estimate the rate of recombination.

**Table 3:** Number of recombination events detected by Rbrothers for several prior values

Prior Expectation (“Lambda_Prior”)	Point Estimate	95% Range
1	50.5	39–56
5	65.8	60–72
10	67.1	61–74
20	79.4	71–88

## Discussion

We sequenced 15 strains of *Ranavirus* collected from *Ambystoma tigrinum* and *A. mexicanum*, collected from field sites and bait shops throughout western North America. Using comparative population genomic analyses, we characterized their diversity, identified potential targets of recent positive selection, and found evidence for recombination. Our results have the following implications: 1) the monophyly of ATV is upheld based on the genealogies constructed from complete genomes of all fully sequenced ranaviruses; 2) although there is evidence that ATV has been spread via recent movement of bait salamanders by humans, our best estimate of its original emergence time is several hundred years ago, before there were roads or a bait trade; 3) eight genes under strong positive selection were identified in ATV (however, the putative function of many of these genes is presently unknown); 4) ATV undergoes recombination, potentially allowing recombination between endemic and bait virus strains.

### Genomic diversity

Although there is large-scale structural variation among *Ranavirus* genomes (Jancovich *et al.* 2010; Mavian *et al.* 2012a; see Figure 3), all of the ATV strains we sequenced were collinear with each other and with the reference ATV strain. Furthermore, there was little diversity in gene content among strains. This lack of diversity may be the result of similar selective forces on gene content—all of the strains sequenced here were collected from *A. tigrinum* in western North America—or the fairly recent origin of ATV, which may not have had time to accumulate gene content differences. Urodeles are known for relatively weak immune responses to viral infection (Cohen 1966; Chen *et al.* 2011); typical ATV die-offs occur in larval populations at ages when individuals cannot yet produce immune globulins (Brunner *et al.* 2004; Greer *et al.* 2008). In addition, a previous microarray study showed that ATV-infected larvae from *A. mexicanum*, the sister taxon of *A. tigrinum*, showed upregulation of genes associated with innate immunity, such as natural killer cells and cytokine signaling, but no appreciable change in expression of genes associated with adaptive immunity (Cotter *et al.* 2008). These implications are supported experimentally, as tiger salamanders are generally susceptible to ATV in infection trials (Storfer *et al.* 2007; Forson and Storfer 2006); susceptibility, however, is dose dependent (Brunner *et al.* 2005). As a result, there may not be strong selection by tiger salamander hosts for ATV to undergo radical shifts in genome structure or co-opt new genes.

Although there was little diversity in gene content, there were three genes (ORF clusters 84, 85, and 88) present in all members of the ATV clade, but absent from all members other strains (see File S1 and Figure 2). These genes are potential adaptations to different host populations in different geographic areas, but none had a well-defined function. Cluster 84 was also identified as being under strong positive selection and showed homology with serum response factor-binding proteins, which are involved in regulation of the cell cycle (reviewed in Rodenberg *et al.* 2010). These three genes are attractive targets for future functional characterization, which may provide insight into the molecular mechanisms of host switches.

### Phylogeography

Ranaviruses have been found in a wide range of cold-blooded vertebrates, and evidence suggests recent host switches among salamanders, turtles, frogs, and fish (Jancovich *et al.* 2010). Jancovich *et al.* (2010) found that ESV and EHNV, which were collected from fish, were more closely related to ATV than the frog or turtle ranaviruses. Our genealogy is consistent with their hypothesis that the common ancestor of the ranaviruses was likely a fish-infecting strain that experienced subsequent host shifts, although ambiguity in the placement of the root resulted in uncertainty about the placement of the ESV/EHNV clade relative to ATV. Jancovich *et al.* (2010) debated whether the common ancestor of the ALRV clade was an amphibian or a fish. Although our data cannot resolve this debate, there is evidence of additional host switching: ADRV, a salamander virus, is most closely related to CMTV, a toad virus (Balseiro *et al.* 2009; Mavian *et al.* 2012a). Note that the presence of recombination in this dataset means that no single genealogy can completely represent the evolutionary history of the samples. However, if recombination events are primarily old or take place between closely related individuals, the topology of the genealogy is expected to reflect the evolutionary history of the majority of the genome (Posada and Crandall 2002). We estimate that the recombination rate is low relative to mutation, and although we only tested for recombination within ATV, we expect that greater sequence divergence and lack of ecological opportunity (*e.g.*, lack of geographic proximity or shared host) limit the amount of recombination between ATV and more distantly related samples. Thus, the conclusion that ATV is monophyletic should be robust to the presence of recombination, and the topology of the genealogies (Figure 2) likely represents the relationships across the majority of the genome.

Our data also support the recent emergence of ATV: the upper limit of the 95% HPD interval for the age of the most recent common ancestor of the ATV strains was < 6000 years (point estimate = 545 years, 95% HPD range = 20–5672 years). The true emergence date likely lies between the most recent common ancestor and our estimated divergence date for ALRV (point estimate = 1526 years, 95% HPD = 53–16,524). Bayesian skyline analysis of all ALRVs (Figure 4) and just ATV (Figure S1) indicate no large changes in population sizes over the last several hundred years, although the confidence intervals are wide. These results suggest that, although recent geographic spread of ATV has resulted from human movement of infected salamanders in the bait trade (Jancovich *et al.* 2005; Picco and Collins 2008), the original emergence of ATV may have happened before such human influence, and any changes in effective population size that might be related to human activity have not yet produced a detectable signal in our data. Note that the emergence date we provide here is based on a small number of strains, all collected within the last 50 years, and so the confidence intervals are quite wide. In addition, recombination between strains can bias estimates of divergence dates: Schierup and Hein (2000) found a downward bias in divergence dates, though this was more pronounced for distance-based phylogeny methods than for maximum-likelihood methods. Thus, it is likely that our estimate of the emergence date of ATV is biased, probably downward. The Bayesian skyline analysis assumes no recombination (Drummond *et al.* 2005), and recombination tends to increase the lengths of the terminal branches of a tree, which can mimic the pattern produced by exponential population growth (Schierup and Hein 2000). Although our inferred rate of recombination is slow (about 1/30 of mutation), and we are not detecting evidence of exponential growth, it is possible that our estimated emergence dates are underestimates, and the effective population size estimates over time may be biased. Nonetheless, this result is consistent with comparative phylogenetic analysis of ATV and its tiger salamander hosts across western North America that showed strong evidence for genealogical concordance, and hence a historical coevolutionary relationship between this host and pathogen (Storfer *et al.* 2007).

### Selection

To identify genes that may be particularly important for adaptation to new hosts or environments, we tested for positive selection using PAML and the MK test. After correcting for multiple comparisons, and removing nonfunctional sequences, eight genes showed evidence for positive selection within the ATV clade. Interestingly, two of these genes are potentially involved in immune response or viral replication: ORF cluster 84 has homology with serum response factor binding proteins, and ORF cluster 95 shows homology to collagen-like proteins, which may shield viral DNA from DNases in the host (Wang *et al.* 2013). ORF cluster 95 is also intriguing because it may be undergoing a process of pseudogenization in some strains, suggesting that it is not essential in all hosts or locations. In addition, the ORFs comprising cluster 95 have substantial genomic overlap with the ORFs comprising cluster 82. ORF cluster 93, a truncated 3-β-hydroxy-D 5-C27-steroid oxidoreductase-like protein (BOH) potentially involved in suppression of host immunity via upregulation of corticosteroids (Chinchar 2002; Delhon *et al.* 2006), is another gene showing evidence of pseudogenization, although after removal of the nonfunctional sequences, it does not appear to be subject to strong selection (note that the sequences included in cluster 93 were validated with Sanger sequencing data, and no errors were detected). In general, however, the genes with the strongest evidence for selection do not have well-characterized functions in the *Ranavirus* genus, so they are interesting targets for future work. It should be noted that the alignments of some of these genes contained substantial indel polymorphism (particularly ORF clusters 66, 84, and 95) that can cause misalignment and an inflated estimate of the number of nonsynonymous mutations. It is also notable that ORF clusters 9 (myristoylated membrane protein; orf53R in FV3), 62 (unknown; orf40R in FV3), and 75 (unknown; orf93L in FV3) were also identified as targets of strong positive selection by Abrams *et al.* (2013) with PAML analyses conducted across all of the ALRV. ORF cluster 9, a myristoylated membrane protein, is required for virion formation in FV3 (Whitley *et al.* 2010). That this gene appears to be a target of adaptive divergence between EHNV/ESV and ATV (indicated by the significant MK test) suggests possible importance for adaptation to new hosts or environments. We note that most ALRV genomes contain another copy of a myristoylated membrane protein (ORF cluster 55) that showed no evidence of positive selection. Similarly, ORF cluster 33 (18K immediate early protein) shows evidence of positive selection and is important for viral replication (Chen *et al.* 2011) (another immediate early protein—cluster 16—also shows evidence for positive selection), as is ORF cluster 1 (subunit a of DNA-dependent RNA polymerase). We also note that many genes may have positive selection on individual codons, but positive selection overall is not strong enough or widespread enough to reject the nearly neutral model across the entire gene.

### Recombination

We provide one of the first tests for recombination among *Ranavirus* strains and the first study to quantify recombination using comparative genomic analyses. We found strong evidence for ongoing recombination within the ATV clade using several methods. Analyses using the RDP4 (Martin *et al.* 2010) and Rbrothers (Irvahn *et al.* 2013) packages, which include nine recombination-detection algorithms, all indicated the presence of recombination among ATV strains. Analyses using LDHat (Auton and McVean 2007) suggest the viral recombination rate, however, is slow (approximately 1/30 the rate of mutation) but sufficient to allow introgression between bait and wild strains. Although LDHat was designed for eukaryotes, and the physical mechanism of recombination in *Ranavirus* is not known, the estimates were very consistent across parameter values. For each putative recombination event, RDP4 attempts to identify a strain similar to the original nonrecombinant sequence (the “major parent”), and a strain similar to the donor of the recombinant sequence (the “minor parent”). It is worth noting that 2 of the 14 best-supported recombination events include potential movement of sequence from bait strains to wild strains (Table S1). However, identification of donor and recipient strains is challenging in a small, closely related sample, and manual examination of the RDP4 results indicates that the events likely represent ancestral recombination that occurred before human movement of salamanders or the evolution of bait strains.

Nevertheless, these results have important implications for understanding the potential for pathogen pollution to affect ranavirus evolution in general and evolution of ATV in particular. Use of *A. tigrinum* as fishing bait has resulted in the movement of ATV strains over large distances, as well as the potential introduction of more virulent strains from bait farms or bait shops into wild populations (Jancovich *et al.* 2005; Storfer *et al.* 2007). Indeed, previous infection trials using bait *vs.* wild strains of ATV (Storfer *et al.* 2007) and FV3 (isolated from wild *vs.* farmed bullfrogs populations; A. Storfer and K. Chojnacki, unpublished data) have shown that bait strains cause greater mortality than native strains in infection trials. As a result, recombination among wild and bait-associated strains has the potential to introduce virulent alleles into locally adapted genomic backgrounds. These concerns extend to other ranavirus species, such as those in commercial aquaculture. Ranaviruses have caused die-offs in hatchery populations of rainbow trout (EHNV) as well as aquaculture populations of pallid sturgeon, European catfish (ECV) and grouper (GIV, SGIV) (Delhon *et al.* 2006), and when asymptomatic fish are used to stock natural populations, new viral strains could be introduced into naïve wild fish populations or recombine with existing strains.

## 
